# Supplementary material for: Benchmarking the translational potential of spatial gene expression prediction from histology
Source: Nat Commun. 2025 Feb 11;16:1544. doi: 10.1038/s41467-025-56618-y (PMC11814321; doi:10.1038/s41467-025-56618-y)
Supplement: Supplementary file 1 — Supplementary Information [file 41467_2025_56618_MOESM1_ESM.pdf]

Supplementary Information

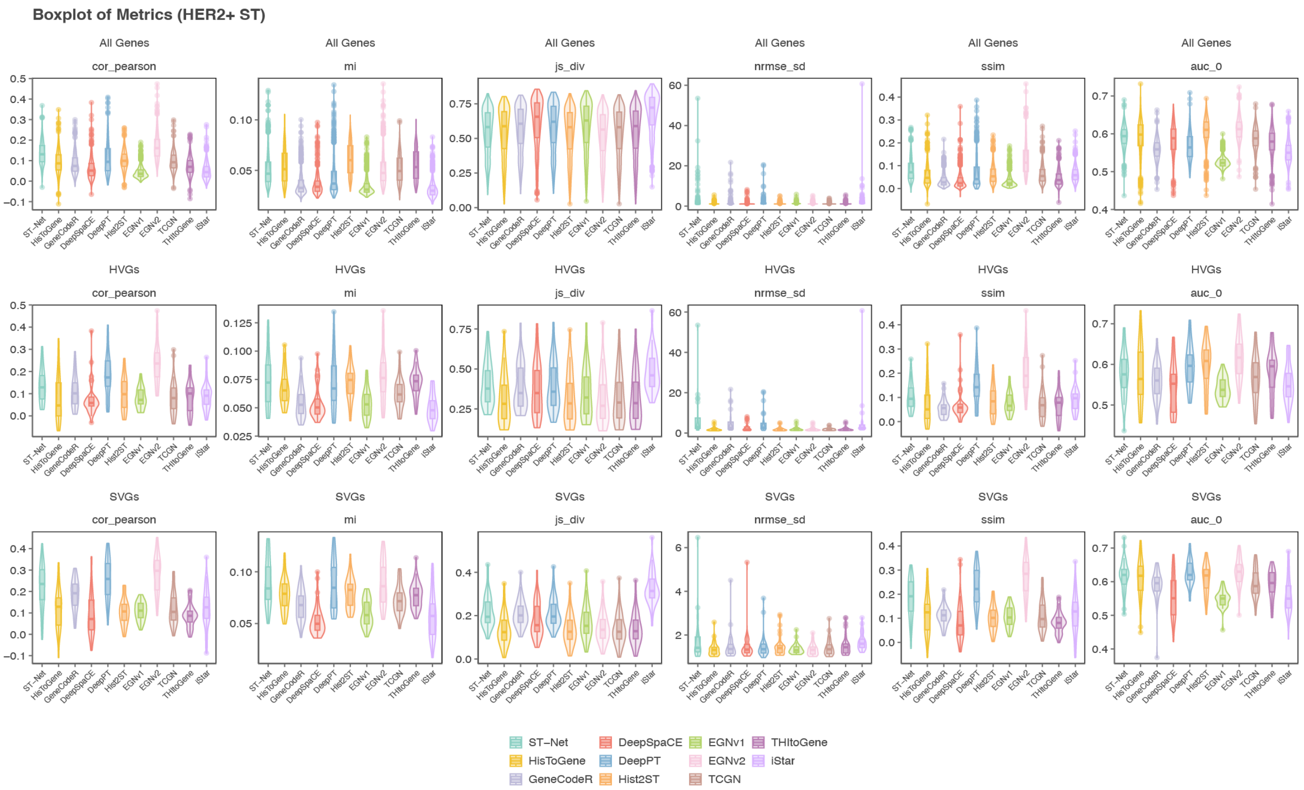

Supplementary Fig. 1: Violin and boxplots of evaluation metrics for gene expression of all genes (*n* = 785), HVGs (*n* = 30) and SVGs (*n* = 20 per image sample) for each method in the HER2+ ST dataset. The bounds of the box correspond to the 25th percentile (first quartile) and 75th percentile (third quartile). The line within the box represents the median. The boxplot's lower whisker extends 1.5 times the interquartile range below the first quartile, while the upper whisker extends 1.5 times the interquartile range above the third quartile. Source data are provided as a Source Data file.

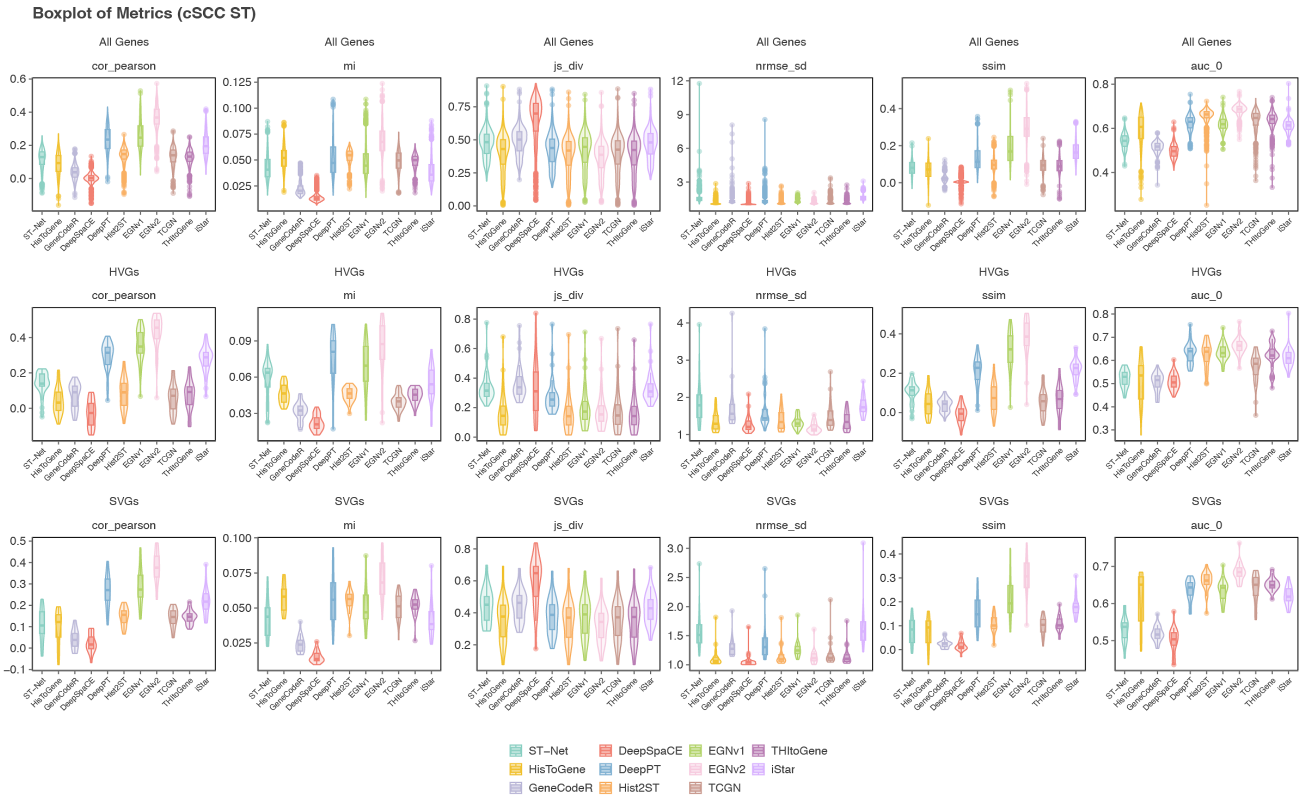

Supplementary Fig. 2: Violin and boxplots of evaluation metrics for gene expression of all genes (n = 997), HVGs (n = 35) and SVGs (n = 20 per image sample) for each method in the cSCC ST dataset. The bounds of the box correspond to the 25th percentile (first quartile) and 75th percentile (third quartile). The line within the box represents the median. The boxplot's lower whisker extends 1.5 times the interquartile range below the first quartile, while the upper whisker extends 1.5 times the interquartile range above the third quartile. Source data are provided as a Source Data file.

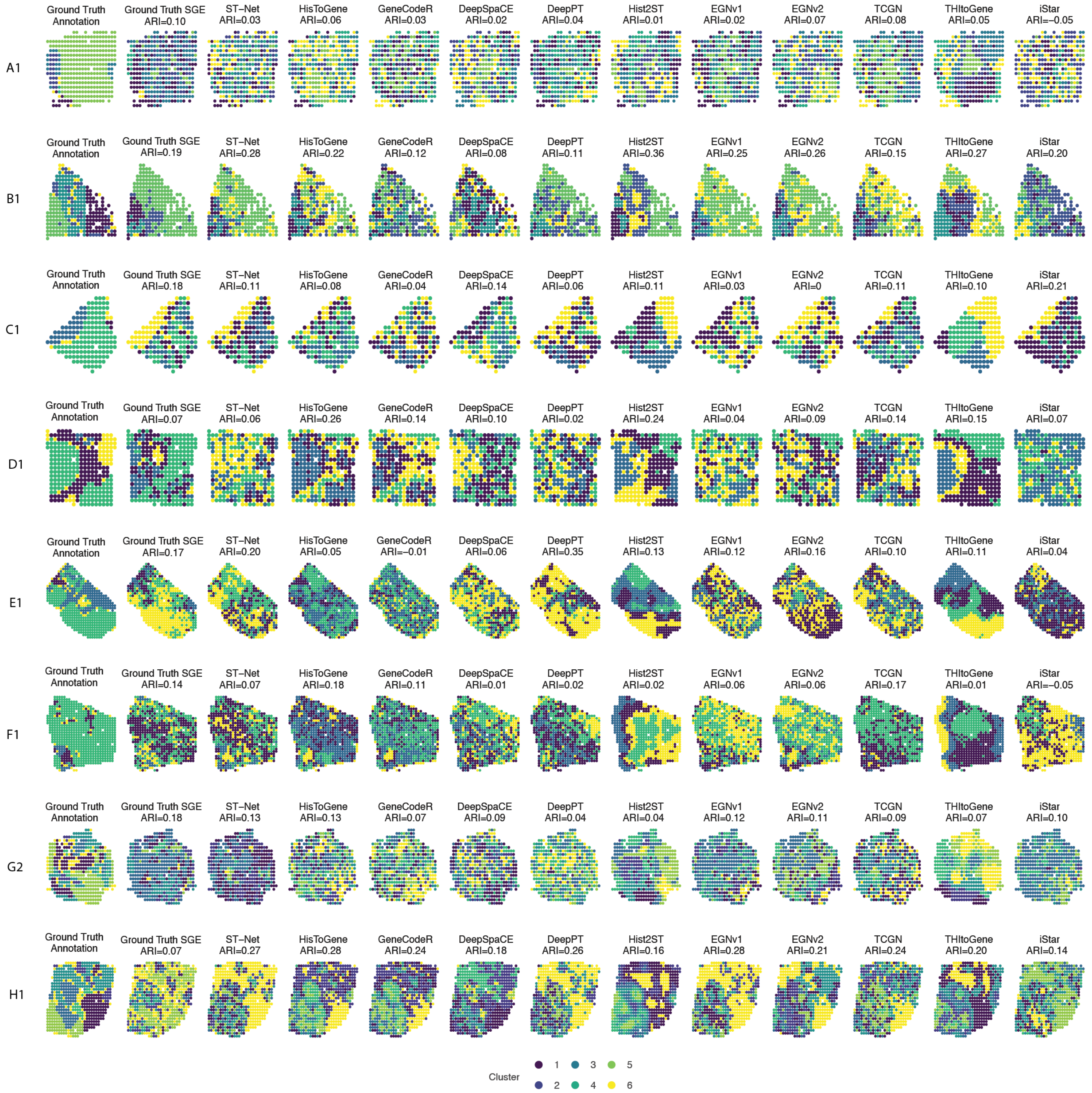

Supplementary Fig. 3: K-means clustering of spatial regions based on eight samples from HER2+ ST dataset was performed using predicted gene expression of each method. The ground truth annotations are based on manual delineation by pathologists, while the ground truth SGE is derived from sequencing data. Adjusted Rand Index (ARI) was calculated between the ground truth annotations and the clustering results of each method. In sample B1, Hist2ST had the best performance, achieving the highest ARI of 0.36, followed by ST-Net with an ARI of 0.28 and THItoGene with 0.27. These methods outperformed the ground truth SGE, which had an ARI of 0.19. Source data are provided as a Source Data file.

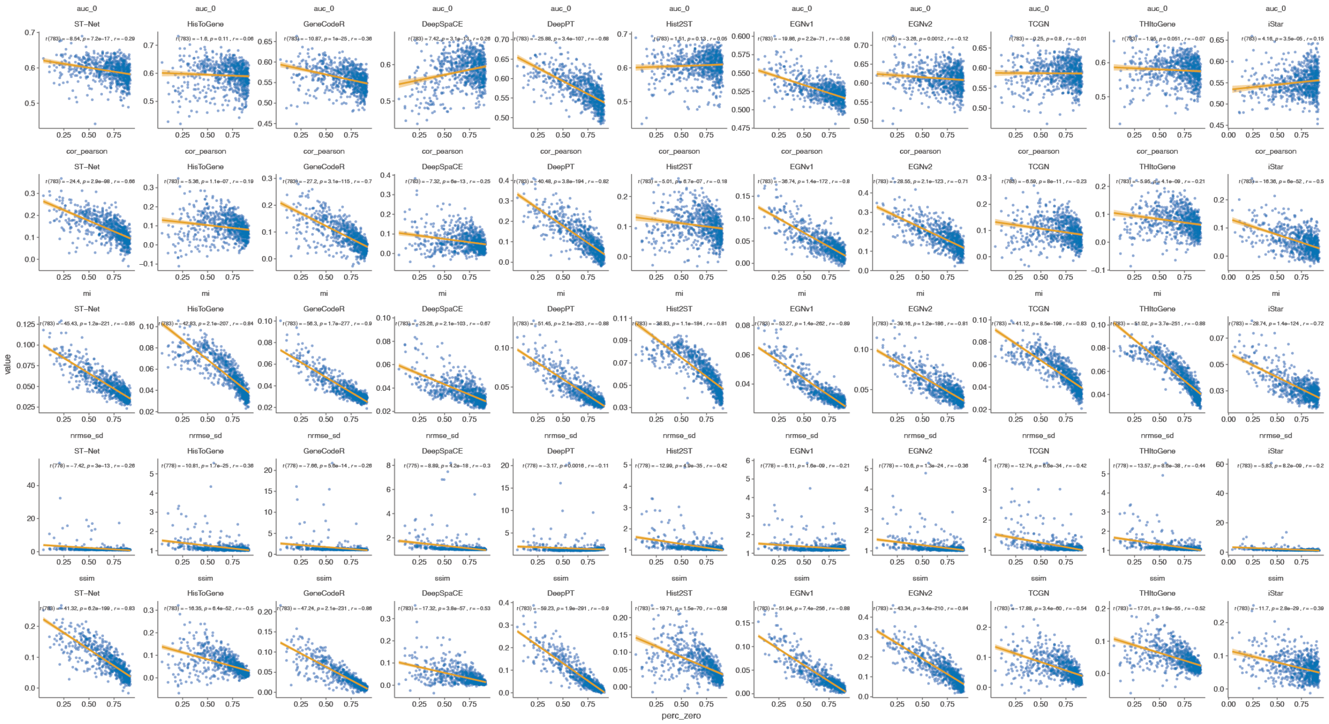

Supplementary Fig. 4: Gene expression prediction evaluation metrics vs. the percentage of zeros in each gene for each method. The solid line indicates the line of best fit, while the error band represents the 95% confidence interval (CI). The test statistics, degrees of freedom, p-values and effect sizes from a correlation test are shown on each panel. Source data are provided as a Source Data file.

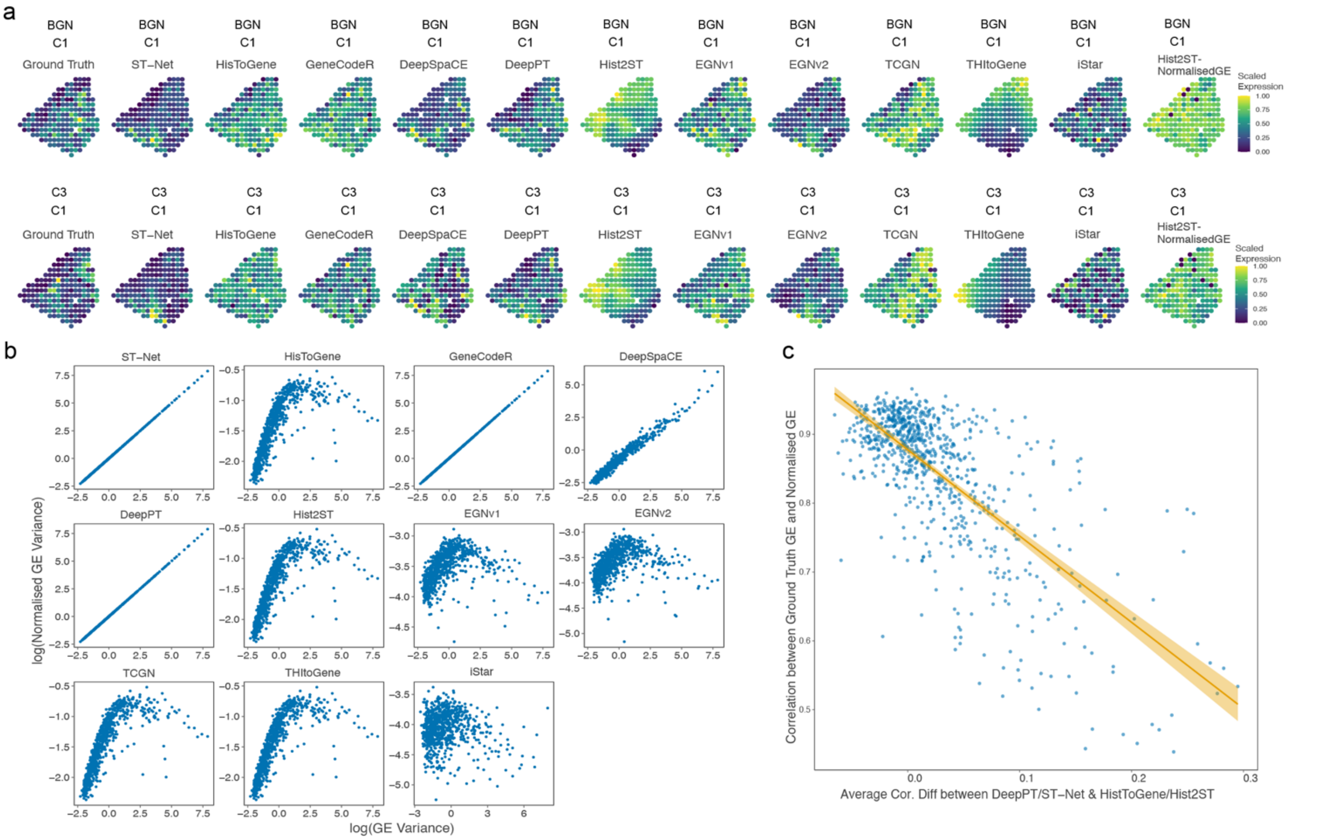

Supplementary Fig. 5: Evaluation of gene expression prediction and normalisation in HER2+ ST dataset. (a) Spatial plots of predicted gene expression of BGN and C3 at each spot in an HER2+ ST image in the test set of each method. Genes were chosen as they had high correlation in DeepPT/ST-Net and low correlation in Hist2ST/HisToGene. Ground truth (leftmost column) and normalised gene expression (rightmost column) values are also plotted. (b) Scatterplot of gene expression variance in the HER2+ ST dataset before (x-axis) and after normalisation (y-axis) for each method. (c) Scatterplot of average correlation difference between average correlation of both DeepPT/ST-Net & average correlation of both HisToGene/Hist2ST and the correlation between ground truth and normalisation (y-axis). Each point represents a gene in the HER2+ ST dataset (*n* = 785). The solid line indicates the line of best fit, while the error band represents the 95% CI. Source data are provided as a Source Data file.

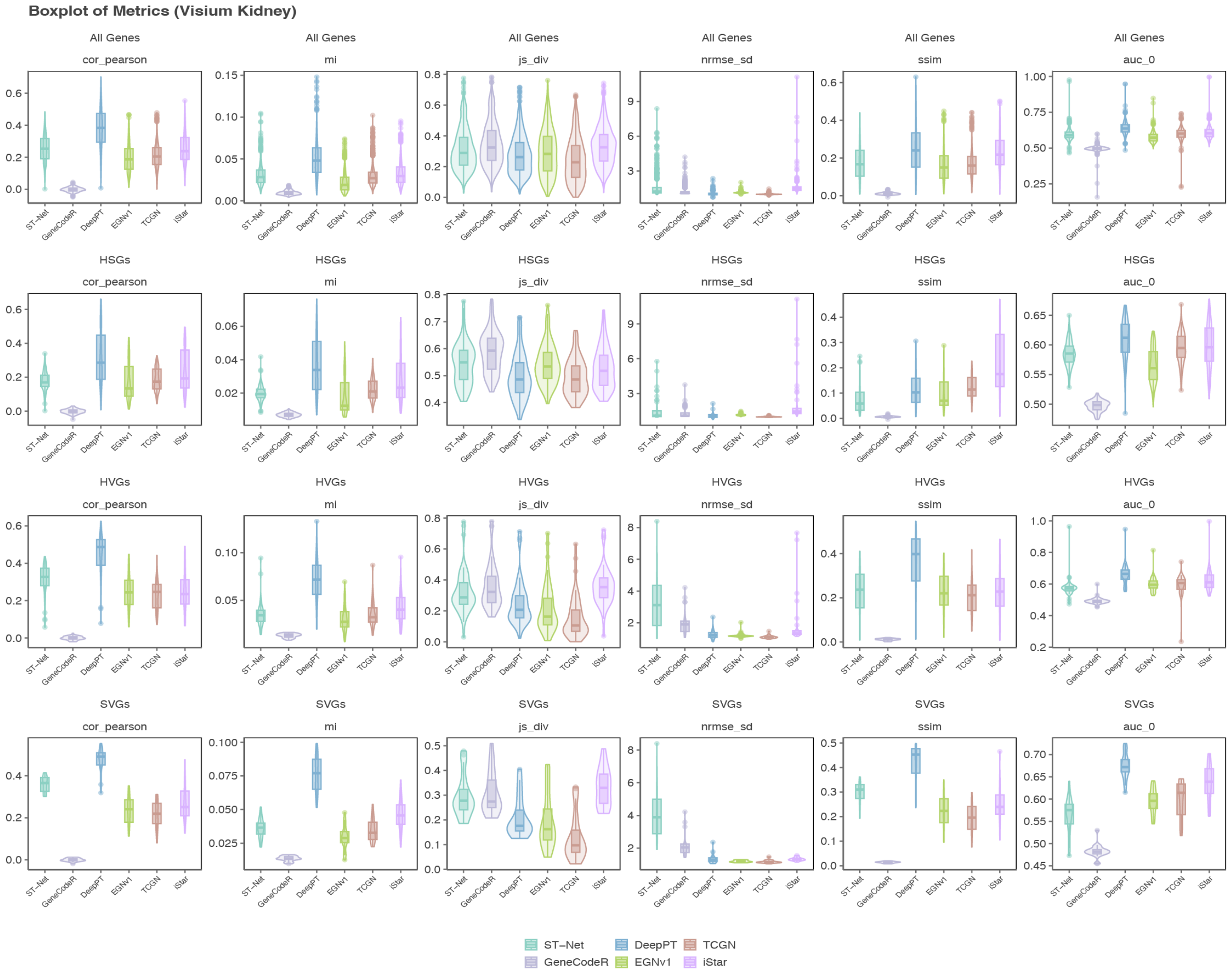

Supplementary Fig. 6: Violin and boxplots of evaluation metrics for gene expression of all genes (n = 992), HSGs (n = 145), HVGs (n = 48) and SVGs (n = 20) for each method in the Visium-Kidney dataset. The bounds of the box correspond to the 25th percentile (first quartile) and 75th percentile (third quartile). The line within the box represents the median. The boxplot's lower whisker extends 1.5 times the interquartile range below the first quartile, while the upper whisker extends 1.5 times the interquartile range above the third quartile. Source data are provided as a Source Data file.

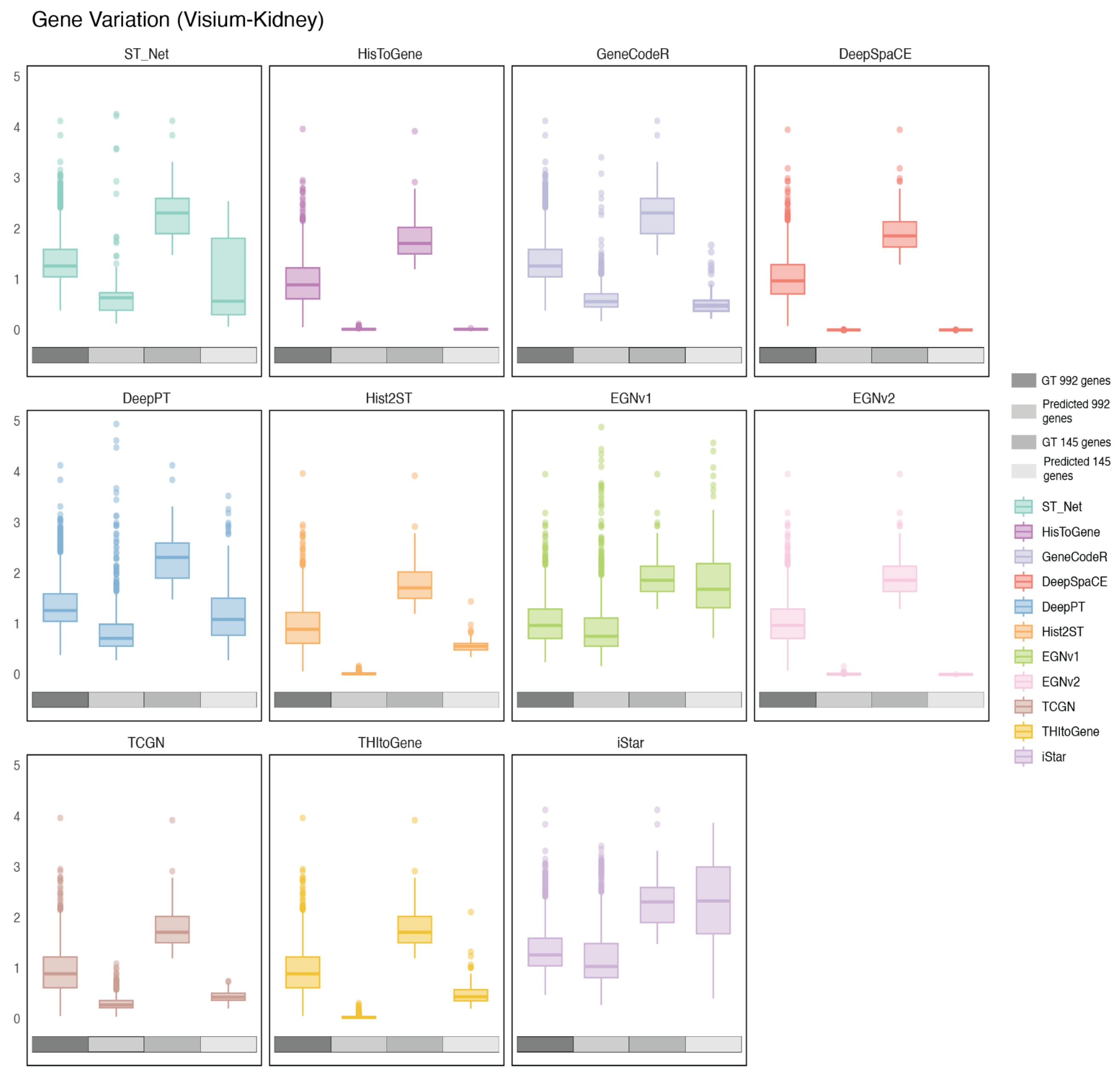

Supplementary Fig. 7: Coefficient of variation for ground truth and predicted 992 genes, and ground truth and predicted 145 HSGs across spots in the Visium-Kidney dataset. The bounds of the box correspond to the 25th percentile (first quartile) and 75th percentile (third quartile). The line within the box represents the median. The boxplot's lower whisker extends 1.5 times the interquartile range below the first quartile, while the upper whisker extends 1.5 times the interquartile range above the third quartile. Outliers are shown as individual data points. Source Data are provided as a Source Data file.

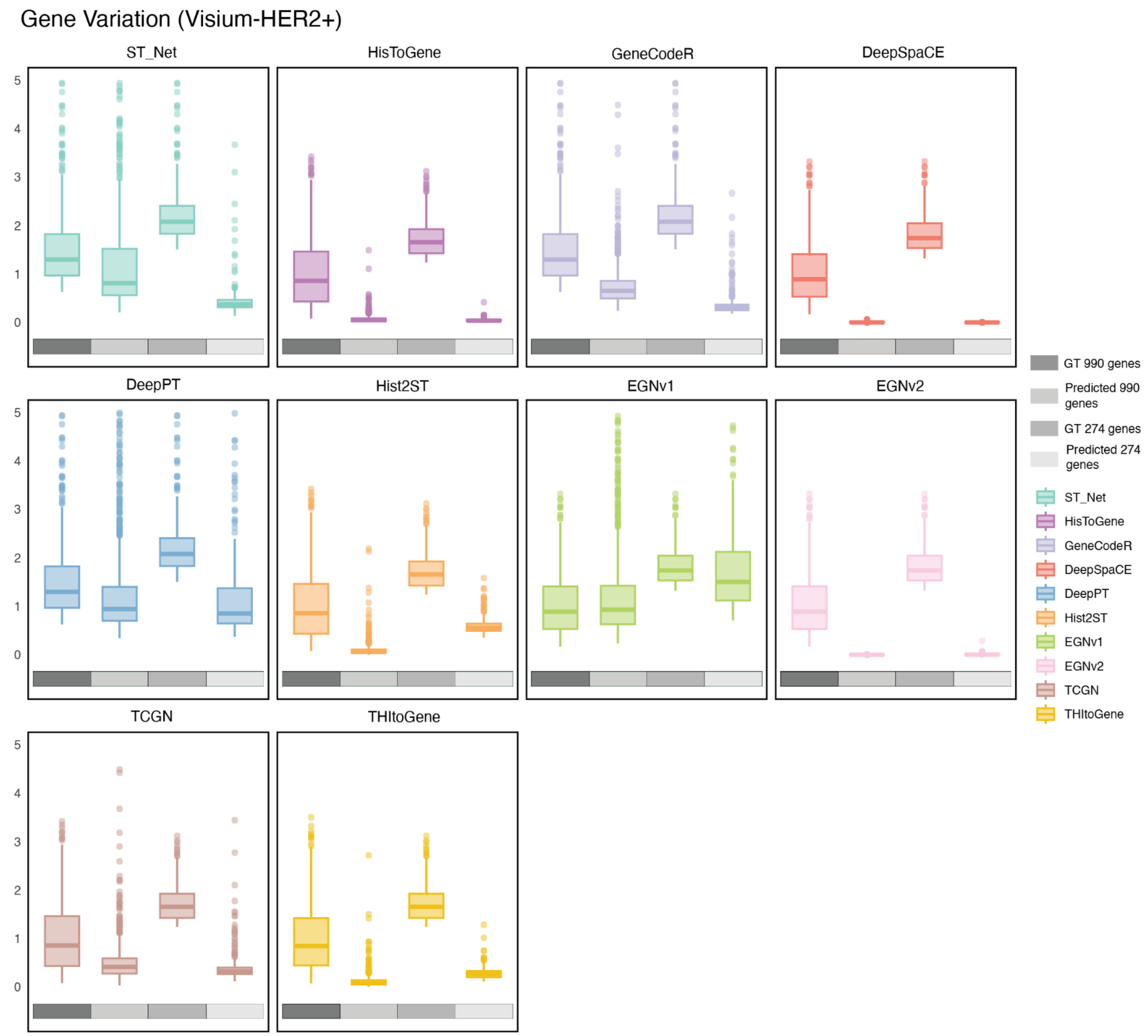

Supplementary Fig. 8: Coefficient of variation for ground truth and predicted 990 genes, and ground truth and predicted 274 HSGs across spots in the Visium-HER2+ dataset. The bounds of the box correspond to the 25th percentile (first quartile) and 75th percentile (third quartile). The line within the box represents the median. The boxplot's lower whisker extends 1.5 times the interquartile range below the first quartile, while the upper whisker extends 1.5 times the interquartile range above the third quartile. Outliers are shown as individual data points. Source data are provided as a Source Data file.

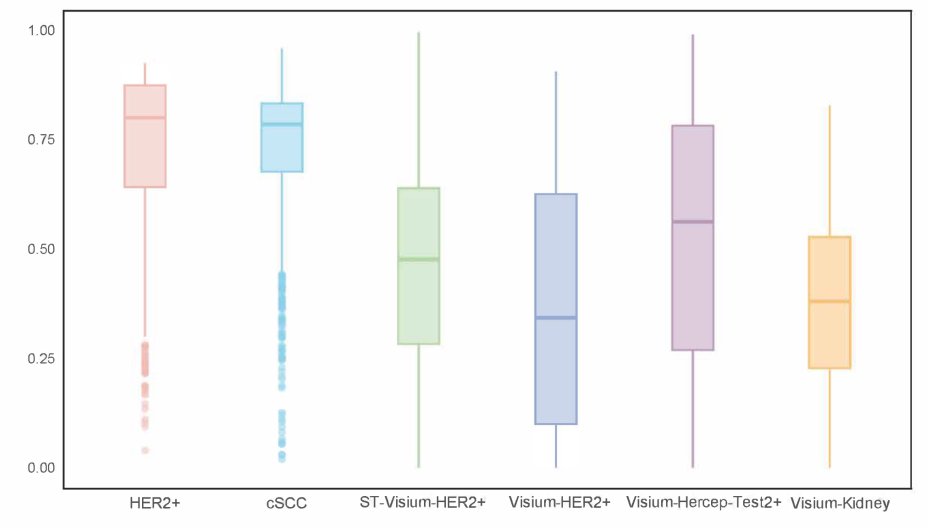

Supplementary Fig. 9: Gene expression matrix sparsity across different datasets. HER2+ (*n* = 785) and cSCC (*n* = 997) refer to the sparsity of the gene sets used for the ST data training and predictions. ST-Visium-HER2+ (*n* = 762) refers to the sparsity of the Visium-HER2+ gene set used for validating HER2+ ST trained models. Visium-HER2+ (*n* = 990) and Visium-Hercep-Test2+ (*n* = 990) represent the sparsity of gene sets used for training and prediction in the Visium Breast Cancer models, respectively. Visium-Kidney (*n* = 992) represents the sparsity of the gene sets in the Visium-Kidney dataset. The bounds of the box correspond to the 25th percentile (first quartile) and 75th percentile (third quartile). The line within the box represents the median. The boxplot's lower whisker extends 1.5 times the interquartile range below the first quartile, while the upper whisker extends 1.5 times the interquartile range above the third quartile. Outliers are shown as individual data points. Source data are provided as a Source Data file.

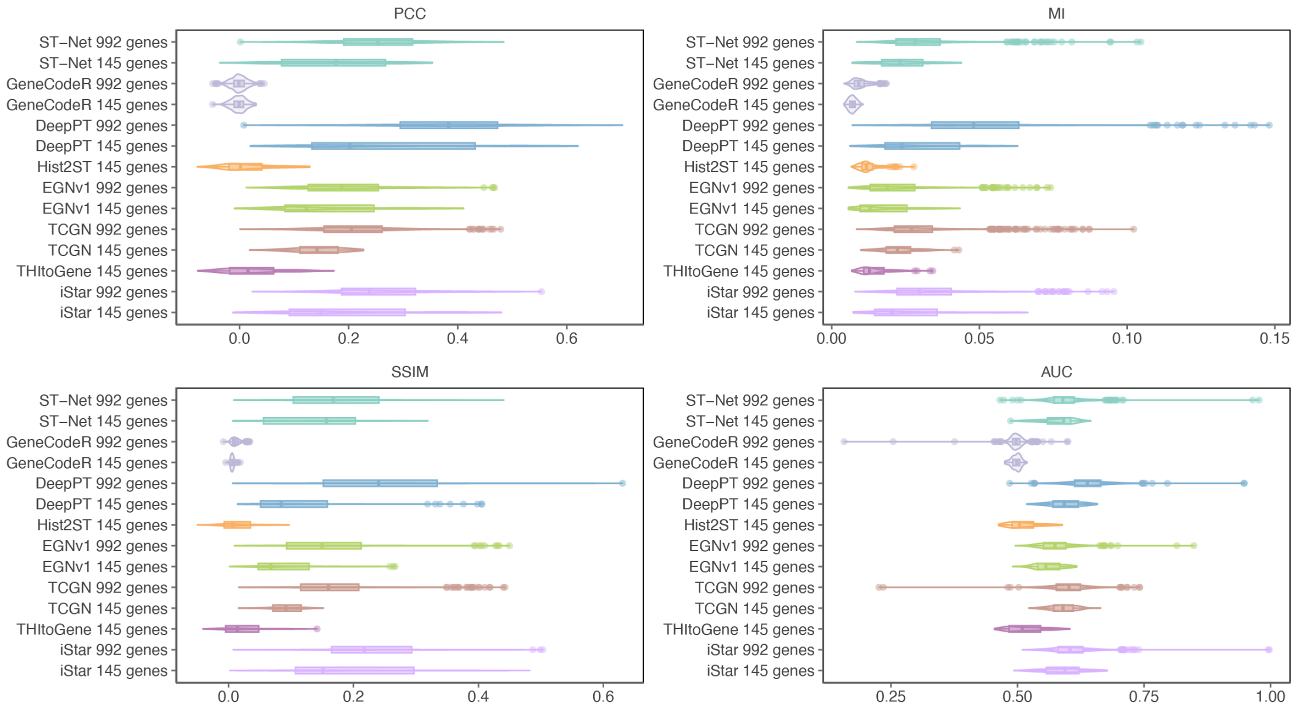

Supplementary Fig. 10: Violin and boxplots of the average PCC, MI, SSIM and AUC between the ground truth gene expression and predicted gene expression. Metrics measured from the test fold of a 4-fold CV, averaged over each gene across all 992 genes and 145 HSGs in the Visium-Kidney dataset. The bounds of the box correspond to the 25th percentile (first quartile) and 75th percentile (third quartile). The line within the box represents the median. The boxplot's lower whisker extends 1.5 times the interquartile range below the first quartile, while the upper whisker extends 1.5 times the interquartile range above the third quartile. Source data are provided as a Source Data file.

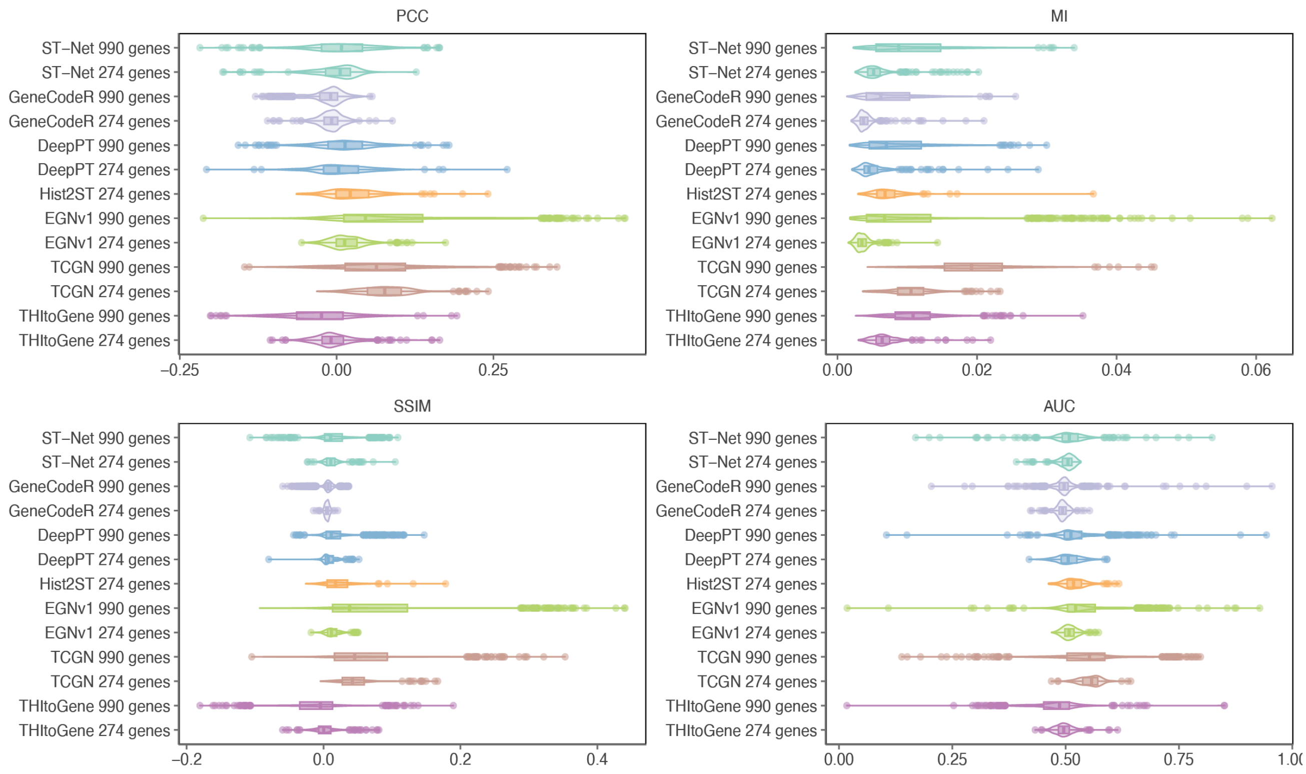

Supplementary Fig. 11: Violin and boxplots of the average PCC, MI, SSIM and AUC between the ground truth gene expression and predicted gene expression. Metrics measured from the test fold of a 4-fold CV, averaged over each gene across all 990 genes and 274 HSGs in the Visium-HER2+ dataset. The bounds of the box correspond to the 25th percentile (first quartile) and 75th percentile (third quartile). The line within the box represents the median. The boxplot's lower whisker extends 1.5 times the interquartile range below the first quartile, while the upper whisker extends 1.5 times the interquartile range above the third quartile. Source data are provided as a Source Data file.

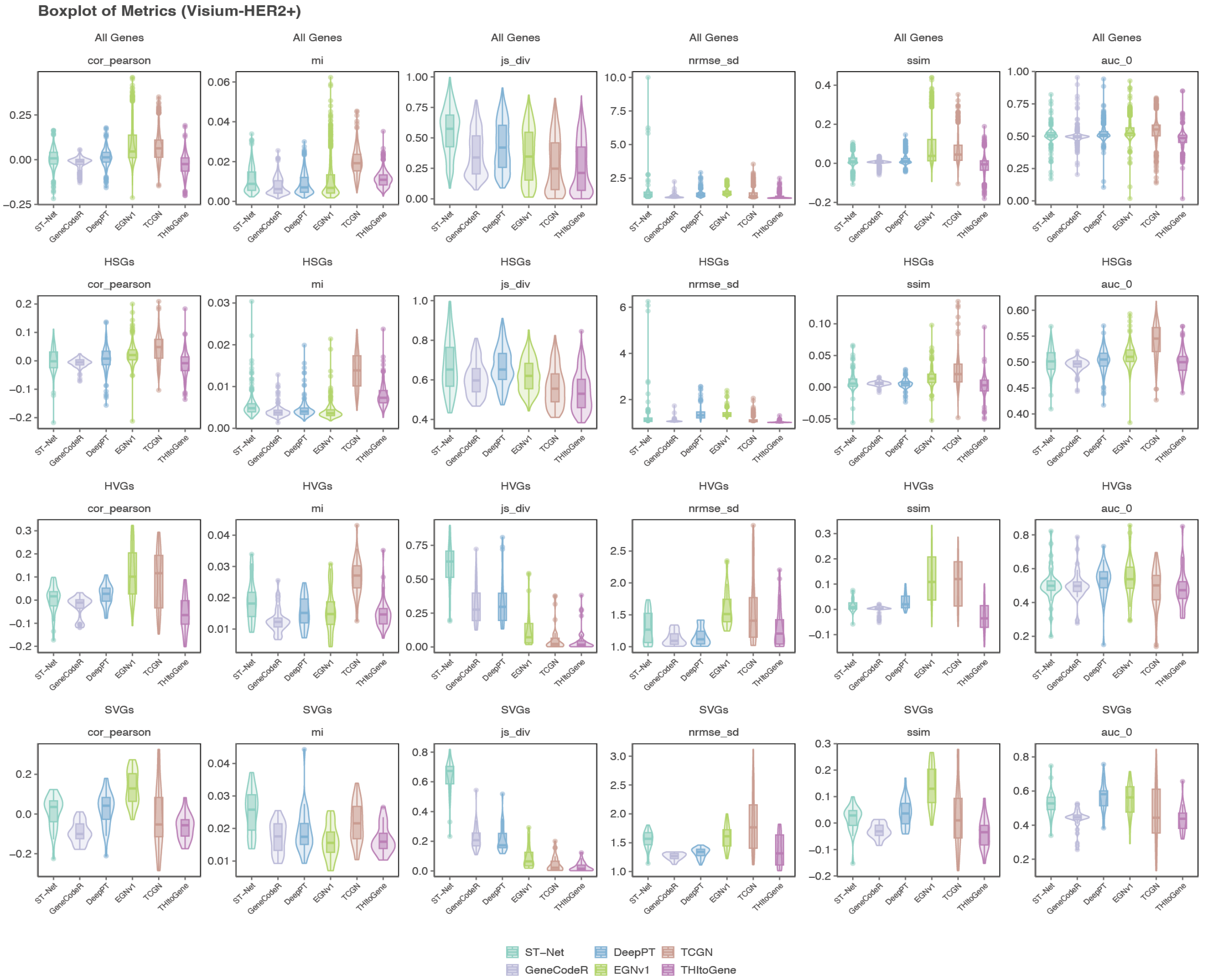

Supplementary Fig. 12: Violin and boxplots of evaluation metrics for gene expression of all genes (n = 990), HSGs (n = 274), HVGs (n = 36) and SVGs (n = 20 per image sample) for each method in the Visium-HER2+ dataset. The bounds of the box correspond to the 25th percentile (first quartile) and 75th percentile (third quartile). The line within the box represents the median. The boxplot's lower whisker extends 1.5 times the interquartile range below the first quartile, while the upper whisker extends 1.5 times the interquartile range above the third quartile. Source data are provided as a Source Data file.

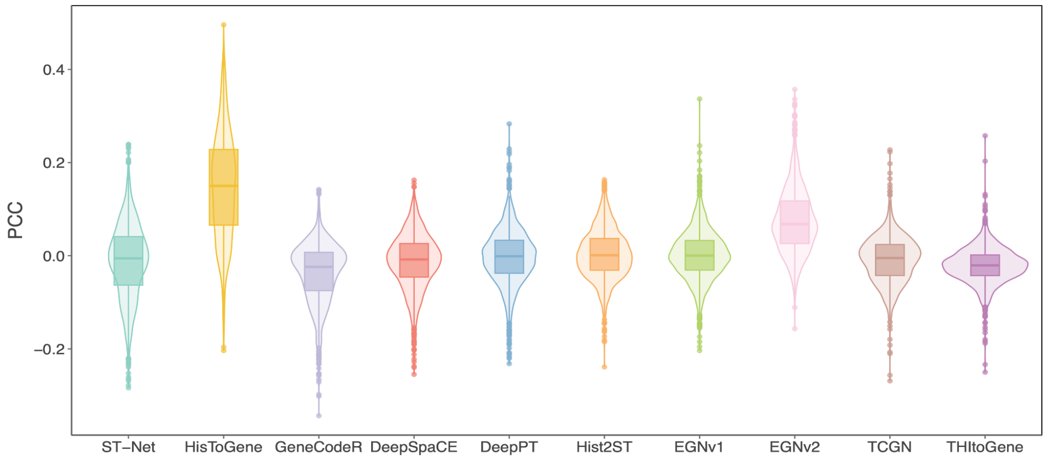

Supplementary Fig. 13: Violin and boxplots of gene-level correlations between ground truth and predicted gene expression (n = 762) across two adjacent tissue slides from Visium-HER2+. The models were trained on the HER2+ ST dataset, with the best-performing model selected based on 4-fold cross-validation. The bounds of the box correspond to the 25th percentile (first quartile) and 75th percentile (third quartile). The line within the box represents the median. The boxplot's lower whisker extends 1.5 times the interquartile range below the first quartile, while the upper whisker extends 1.5 times the interquartile range above the third quartile. Source data are provided as a Source Data file.

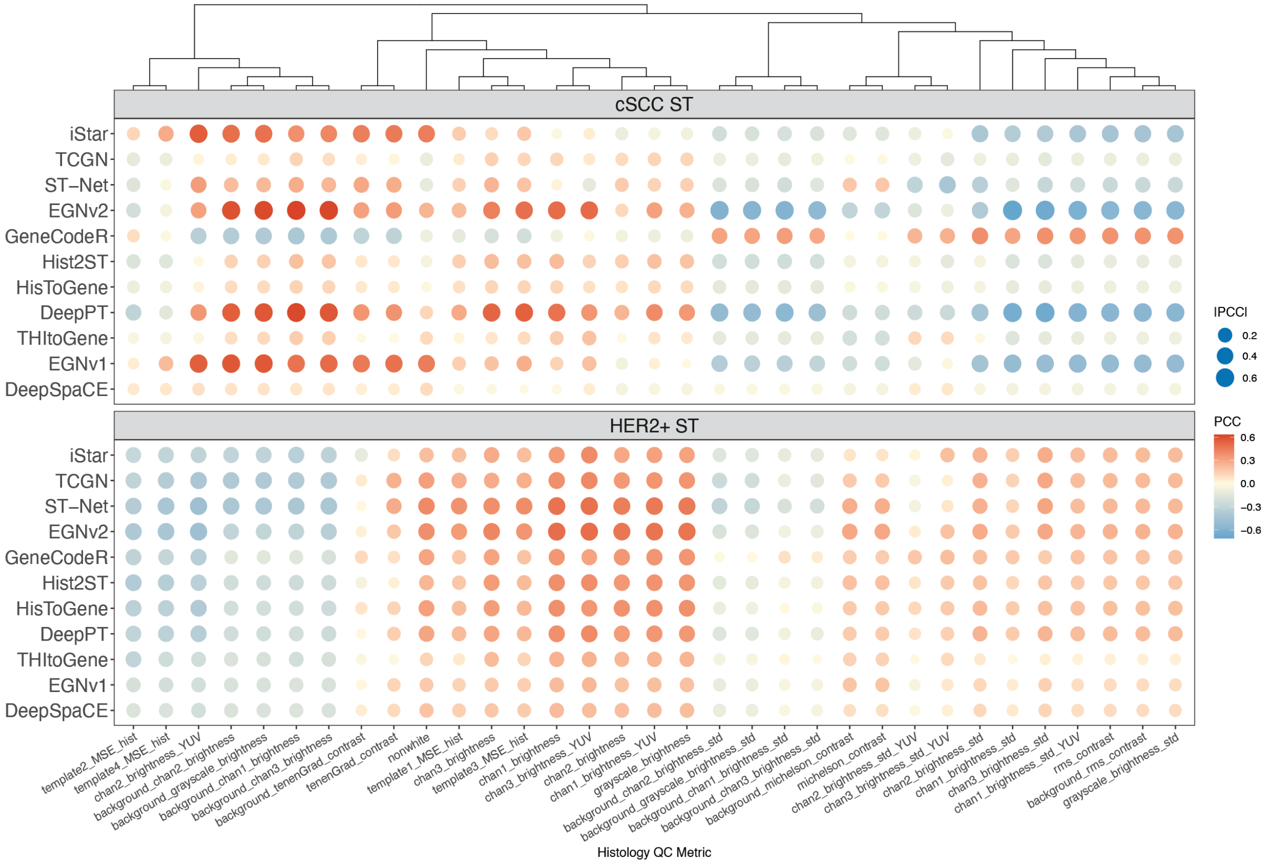

Supplementary Fig. 14: Dotplot of correlation between various histology QC metrics and gene-level correlations for each method in the HER2+ ST dataset and the cSCC ST dataset. Source data are provided as a Source Data file.

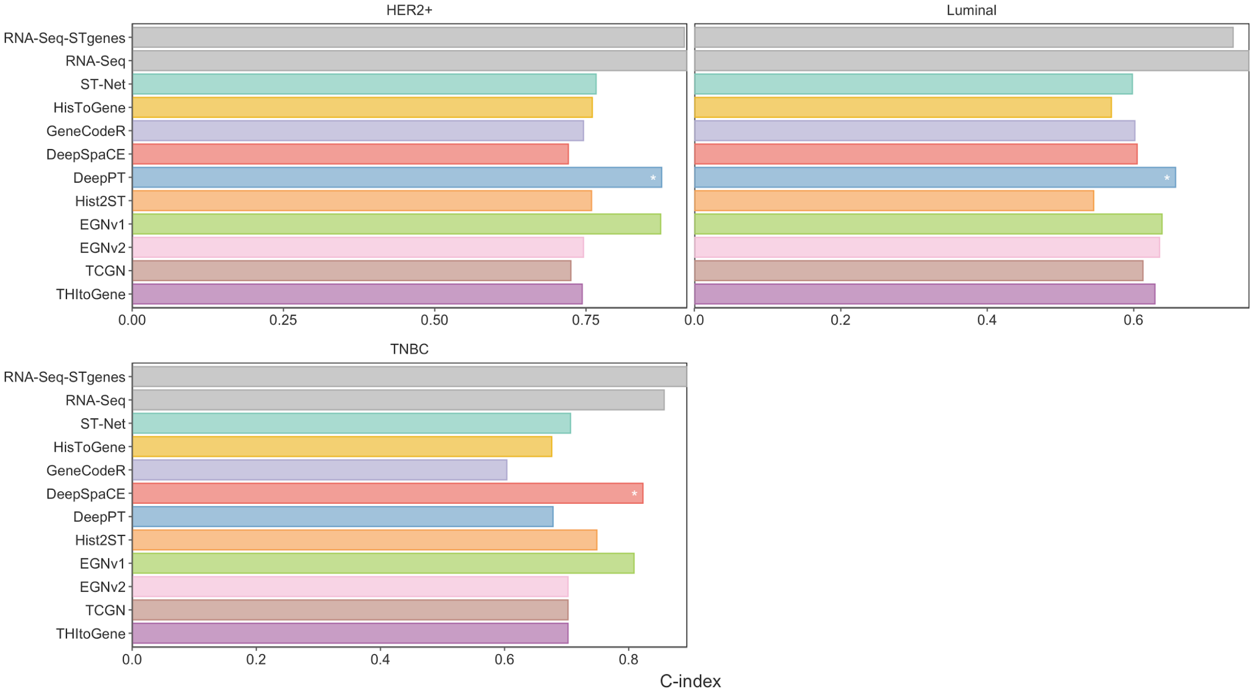

Supplementary Fig. 15: C-indices of multivariate cox regression models predicting survival of TCGA-BRCA patients, using RNA-Seq bulk, RNA-Seq bulk using only genes present in HER2+ ST dataset, and the predicted pseudobulk from each method. C-indices were calculated from the training data of models trained within HER2+ (*n* = 92), luminal (*n* = 463) and TNBC (*n* = 79) breast cancer clinical subtypes. Source data are provided as a Source Data file.

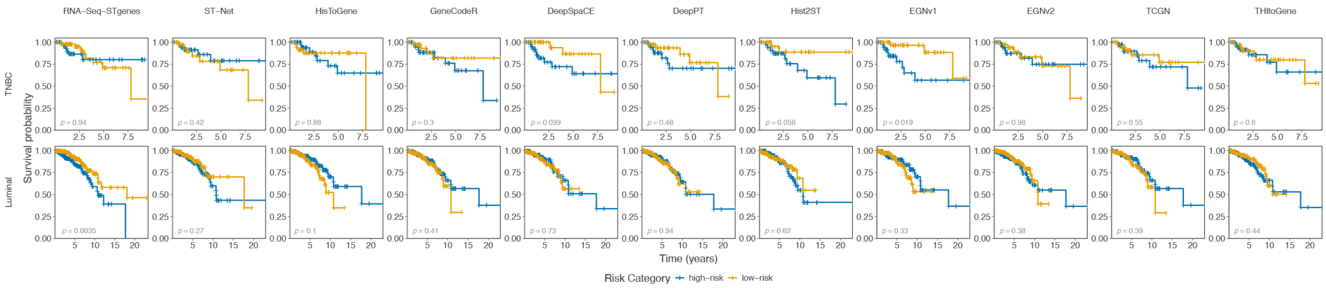

Supplementary Fig. 16: Kaplan-Meier curves for patients split into high and low risk groups by the median risk prediction of the multivariate cox regression models for each method in luminal (*n* = 463) and TNBC (*n* = 79) breast cancer subtypes. The average risk prediction from a 3-fold CV with 100 (*n* = 100) repeats was used. The p-value represents the result of the two-sided log-rank test for assessing the statistical significance of differences in survival between the groups. Source data are provided as a Source Data file.

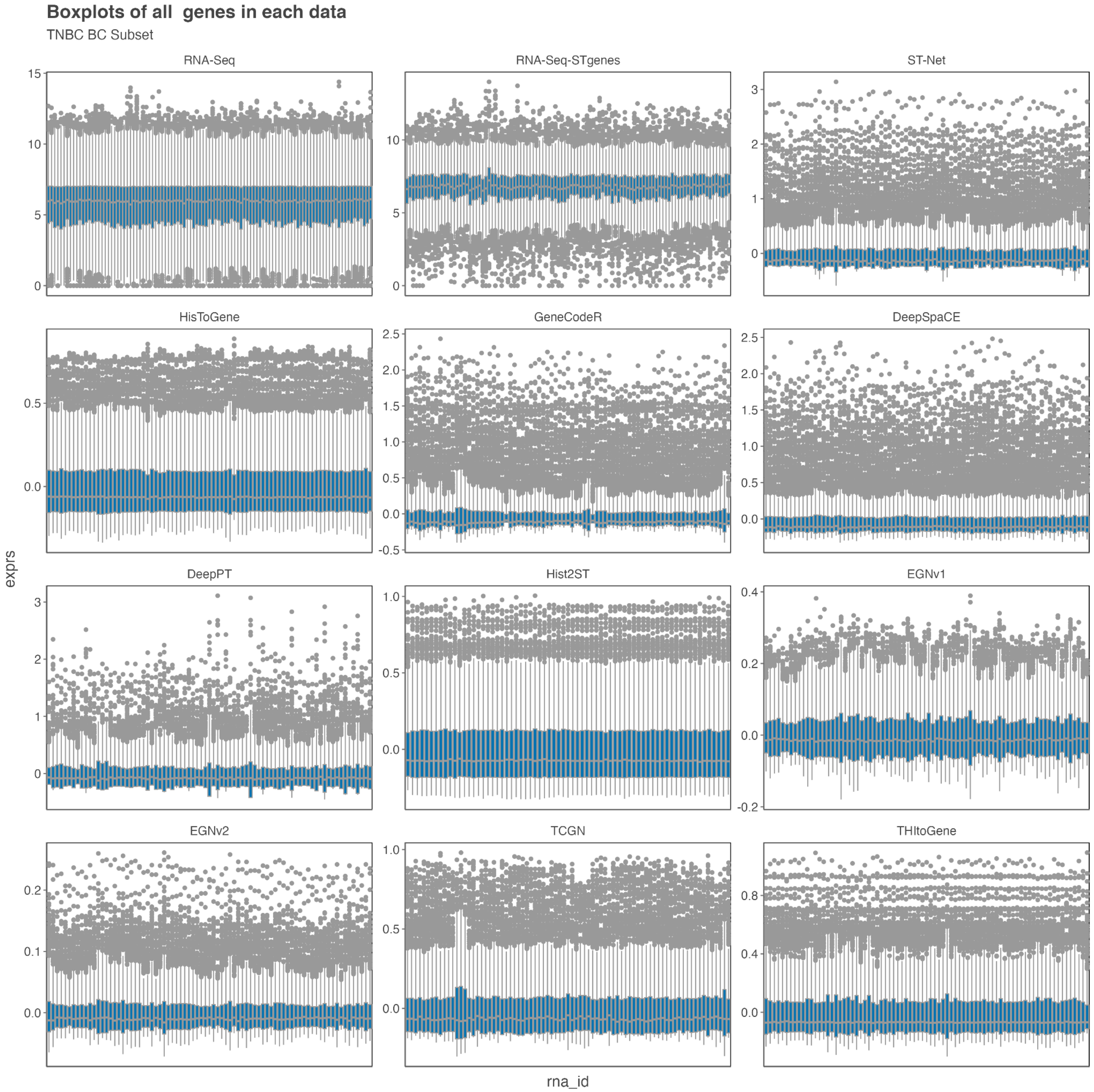

Supplementary Fig. 17: Boxplot of gene expression values after transformation (n = 15957) for each sample from the TNBC subset (n = 79) of the TCGA data and for all genes (n = 749) that were presented in the HER2+ ST dataset. The bounds of the box correspond to the 25th percentile (first quartile) and 75th percentile (third quartile). The line within the box represents the median. The boxplot's lower whisker extends 1.5 times the interquartile range below the first quartile, while the upper whisker extends 1.5 times the interquartile range above the third quartile. Outliers are shown as individual data points. Source data are provided as a Source Data file.

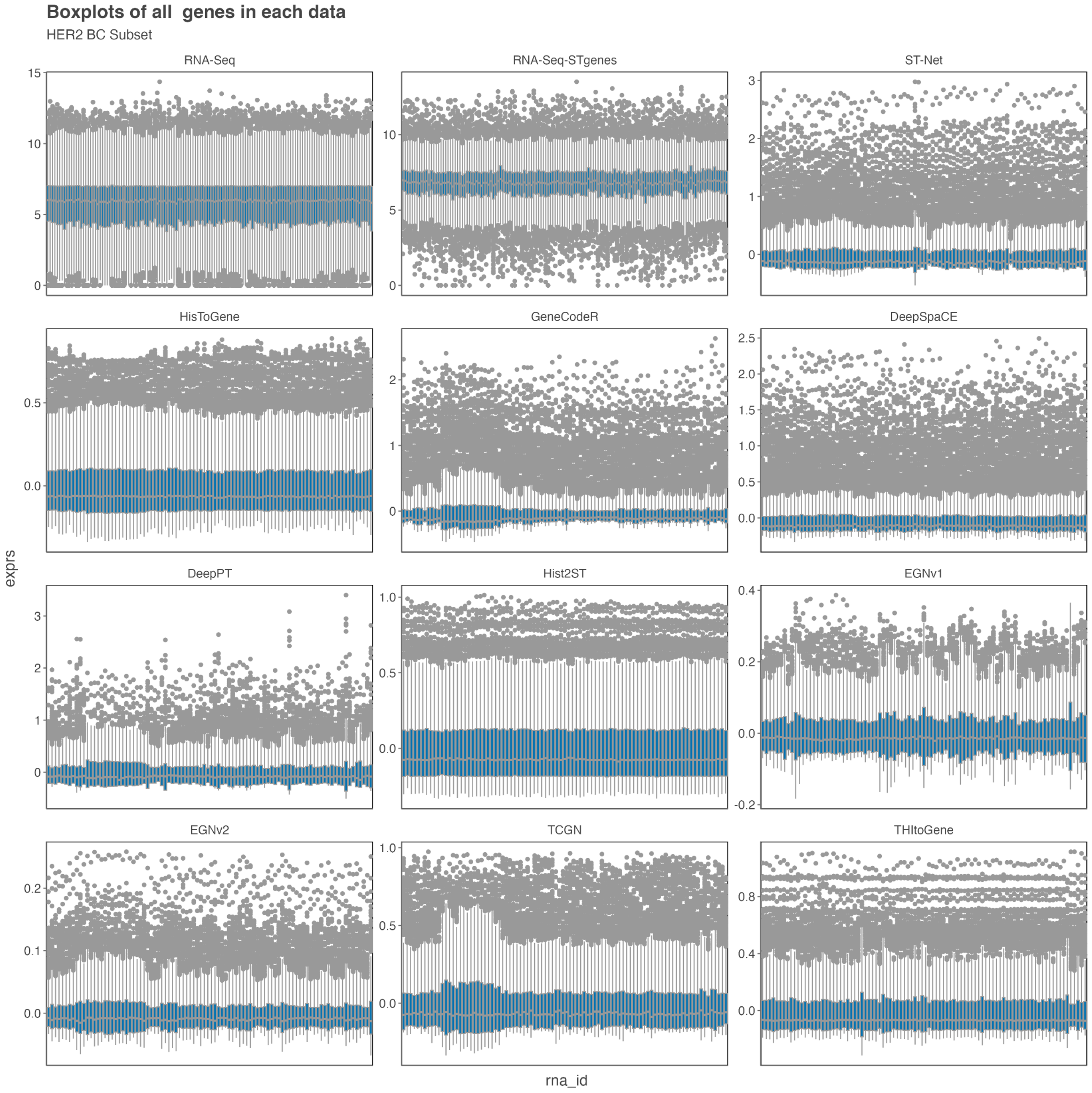

Supplementary Fig. 18: Boxplot of gene expression values after transformation (*n* = 15957) for each sample from the HER2 subset (*n* = 92) of the TCGA data and for all genes (*n* = 749) that were presented in the HER2+ ST dataset. The bounds of the box correspond to the 25th percentile (first quartile) and 75th percentile (third quartile). The line within the box represents the median. The boxplot's lower whisker extends 1.5 times the interquartile range below the first quartile, while the upper whisker extends 1.5 times the interquartile range above the third quartile. Outliers are shown as individual data points. Source data are provided as a Source Data file.

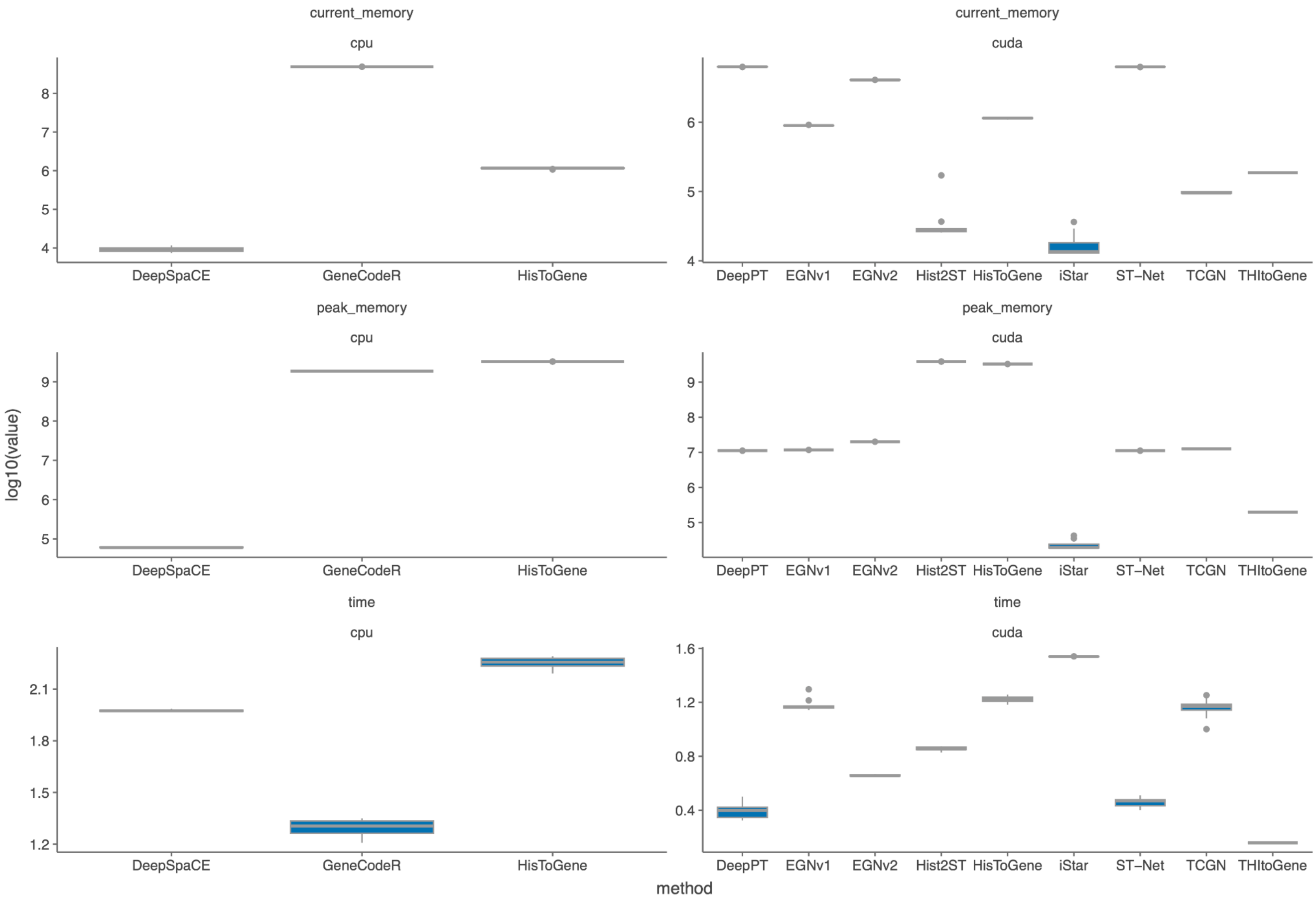

Supplementary Fig. 19: Boxplots of the computational efficiency of methods when trained on one histology image using 10 epochs. Metrics were measured for parallelised (cuda) and non-parallelised (cpu) training where applicable. Memory was measured in bytes and time in seconds. The bounds of the box correspond to the 25th percentile (first quartile) and 75th percentile (third quartile). The line within the box represents the median. The boxplot's lower whisker extends 1.5 times the interquartile range below the first quartile, while the upper whisker extends 1.5 times the interquartile range above the third quartile. Outliers are shown as individual data points. Source data are provided as a Source Data file.

Supplementary Table 1: Summary of Methods predicting SGE from H&E

Model	Local Features (one spot)	Local + Global Features (spot-neighbourhood relations)	Global Features (spot-spatial relations)	Reference Dataset	Reference Encoder	Application method
ST-Net	Pretrained DenseNet 121	NA	NA	NA	NA	NA
HisToGene	Learnable Linear Layer	Super Resolution	ViT	NA	NA	NA
DeepPT	Pretrained ResNet50 + Autoencoder + MLP	NA	NA	NA	NA	NA
Hist2ST	Convmixer	GNN - GraphSAGE	Transformer	NA	NA	NA
DeepSpaCE	VGG16	Super Resolution	NA	NA	NA	NA
GeneCodeR	Non deep learning method					
EGNv1	ViT + Exemplar	NA	Exemplar	Internal Dataset	ResNet50	Exemplar Retrieval
EGNv2	Exemplar (ResNet) + GraphSAGE + GCN	NA	Exemplar	Internal Dataset	ResNet18	Exemplar Retrieval
XFuse	Statistical Model + Deep generative model			ISC Data	NA	Fuse with H&E
BLEEP	Pretrained ResNet50	NA	Contrastive Learning	Internal Dataset	ResNet50 and FCN	Contrastive Learning
NSL	Stain deconvolution matrix					
TCGN	CNN + ViT + GNN	NA	NA	NA	NA	NA
BrST-Net	Trained 10 state-of-the-art CNN models and transformers then compared their performances + introduced an auxiliary network					
TransformerST	CNN + cross-scale internal GNN	Adaptive Graph Transformer	Conditional Transformer	NA	NA	NA
STimage	Pretrained ResNet50 + Negative Binomial	NA	NA	NA	NA	NA
THItoGene	Dynamic Convolution + Efficient-Capsule Module	Graph Attention Network (GAT)	ViT	NA	NA	NA
SEPAL	Image Encoder	GNN	NA	NA	NA	NA
iStar	HViT (super resolved features at near single-cell level)	NA	HViT	NA	NA	NA

Supplementary Table 2: Summary of Methods advantages, limitations, user guidance and improvement directions

Model	Date of Publication	Publication	Advantages	Limitations	User Guidance	Improvement Directions
ST-Net	22/06/2020	Nature Biomedical Engineering	Simple model, straightforward implementation, consistent performance across ST and Visium data	Low generalizability and clinical translational impact	Serve as baseline	Using encoder pre-trained on larger and more diverse tissue datasets
HisToGene	28/11/2021	Pre-print	Suitable for handling small sample size, simple implementation, relatively high generalizability	Modest performance on SGE prediction and clinical translational impact, need to carefully set the image and position embedding dimensions, hard to train on Visium data	Use for small, labelled datasets or directly apply trained model to external datasets for SGE prediction for baseline comparison	Reduce model complexity, particularly for patch embedding, try multi-scale modules that better handle super-resolution
GeneCodeR	16/01/2022	Pre-print	Fast training and interpretability for R users	Modest performance	Explore spatial information from images to gene expression	Consider more advanced statistical model and perform pre-processing of the H&E image stain to reduce patient effect
DeepSpaCE	08/03/2022	Scientific Reports	Simple implementation, relatively high generalizability and clinical translational impact	Modest performance on SGE prediction, hard to train on Visium data	Directly apply trained model to external datasets for SGE prediction as well as survival predictions for baseline comparison	Reduce model complexity, replacing with more efficient modules for feature extraction
DeepPT	09/06/2022	Pre-print	Simple model, simple implementation, performing relatively well on both ST and Visium data	Low generalizability	Serve as baseline	Using encoder pre-trained on larger and more diverse tissue datasets
Hist2ST	20/09/2022	Briefings in Bioinformatics	Perform relatively well in TCGA survival analysis	Complex model, need to carefully set the image and position embedding dimensions, hard to train on Visium data	Apply to survival analysis and served as a baseline	Reduce model complexity, improve global feature representations

EGNv1	26/02/2023	WACV	Potentially less data to train, performing relatively well on both ST and Visium data, enhanced robustness and relatively high clinical translational impact	Requires well-curated, diverse reference dataset, exemplar increase the overall computational cost for large reference data, need to modify the dataloader appropriately to handle new inputs	Use when well-curated, high quality reference data is available, potentially can be used to explore survival analysis	Using encoder pre-trained on larger and more diverse tissue datasets, involve more relevant and representative reference data
EGNv2	26/09/2023	Pattern Recognition	Potentially less data to train, enhanced robustness, relatively good performance in the ST datasets	Requires well-curated, diverse reference dataset, exemplar and graph construction is time-intensive for large reference data, need to modify the dataloader appropriately to handle new inputs, hard to train on Visium data	Use when well-curated, high quality reference data is available, can be used to explore survival analysis - especially luminal breast cancer	Using encoder pre-trained on larger and more diverse tissue datasets, involve more relevant and representative reference data
TCGN	25/11/2023	Medical Image Analysis	Capture cell organisation features (cell-cell interaction) using GNN, works better on Visium than ST data	Complex model, taking relatively more time to train	Explore and understand the effectiveness of transformer and GNN	Reduce model complexity, improve global feature representations
THItoGene	25/12/2023	Briefings in Bioinformatics	Extract deep, multi-view neighbourhood features	Complex model, risk of overfitting, hard coded dataloader and model settings, hard to train on Visium	Explore and understand the effectiveness of deep neighbourhood features	Improve the integration of local and global neighbourhood features
iStar	02/01/2024	Nature Biotechnology	Enable near single-cell SGE prediction using spot-based data	Infeasible to perform training and prediction across different datasets due to both image and spot resolution difference and the requirement of consistent feature embedding dimension, the published code needs to be modified to dynamic dataloader to train more whole H&E embedding at one time	Explore finer single-cell spatial features even when only spot-based SGE data is available, works better on high-resolution image data	Improve spot to single-cell gene expression prediction method, which better allocates spot-based gene expression to the large number of cells within spots

Supplementary Table 3: Top 20 predicted genes by correlation in HER2+ ST data.

gene	EGN v2	Deep PT	Deep Spa CE	Gene Code R	HisTo Gene	Hist 2ST	ST-Net	EGN v1	TCGN	THIto Gene	iStar	overal l_mea n_cor
GNAS	**0.47**	0.41	0.38	0.29	0.35	0.26	0.28	0.19	0.30	0.23	0.26	0.31
FASN	**0.46**	0.40	0.32	0.28	0.31	0.21	0.37	0.18	0.29	0.20	0.28	0.30
SCD	**0.42**	0.34	0.24	0.15	0.28	0.22	0.31	0.13	0.27	0.21	0.21	0.25
MYL12B	**0.33**	0.30	0.23	0.19	0.28	0.26	0.27	0.13	0.24	0.22	0.15	0.24
CLDN4	**0.36**	0.31	0.2	0.23	0.28	0.21	0.27	0.13	0.25	0.16	0.18	0.24
FN1	**0.38**	0.26	0.18	0.17	0.27	0.24	0.20	0.15	0.18	0.22	0.17	0.22
RHOB	**0.30**	0.29	0.25	0.19	0.25	0.25	0.21	0.10	0.21	0.21	0.11	0.22
STMN1	**0.33**	0.28	0.24	0.15	0.26	0.23	0.22	0.11	0.20	0.18	0.17	0.21
HLA.DRA	**0.37**	0.36	0.13	0.21	0.19	0.18	0.23	0.17	0.17	0.12	0.13	0.20
TMBIM6	**0.33**	0.28	0.14	0.17	0.23	0.20	0.25	0.10	0.16	0.18	0.17	0.20
TMEM123	**0.32**	0.27	0.13	0.16	0.24	0.22	0.21	0.10	0.20	0.18	0.17	0.20
CCT4	**0.28**	0.27	0.19	0.14	0.25	0.22	0.23	0.09	0.20	0.19	0.13	0.20
FADS2	**0.32**	0.27	0.10	0.26	0.21	0.15	0.25	0.12	0.19	0.14	0.15	0.20
NDUFB2	**0.28**	0.26	0.14	0.18	0.24	0.25	0.22	0.10	0.17	0.21	0.10	0.20
PRKCSH	**0.31**	0.30	0.11	0.23	0.21	0.16	0.26	0.11	0.17	0.12	0.14	0.20
HMGB2	**0.30**	0.24	0.23	0.13	0.23	0.23	0.21	0.07	0.19	0.18	0.12	0.19
CRACR2B	**0.30**	0.26	0.14	0.20	0.21	0.16	0.26	0.09	0.22	0.13	0.15	0.19
SRSF1	**0.30**	0.26	0.20	0.14	0.23	0.21	0.23	0.08	0.22	0.17	0.09	0.19
CD74	**0.34**	0.38	0.21	0.18	0.05	0.05	0.31	0.17	0.17	0.01	0.20	0.19
HNRNPUL2	**0.29**	0.27	0.17	0.20	0.21	0.18	0.22	0.10	0.19	0.14	0.01	0.19

Supplementary Table 4: Top 20 predicted genes by correlation in cSCC ST data.

gene	EGN v2	Deep PT	Deep Spa CE	Gene Code R	HisTo Gene	Hist2 ST	ST-Net	EGN v1	TCGN	THIto Gene	iStar	overall_mean_cor
PFN1	**0.53**	0.41	0.08	0.12	0.12	0.27	0.13	0.49	0.21	0.23	0.32	0.26
RPL9	**0.52**	0.36	0.06	0.07	0.16	0.24	0.16	0.50	0.22	0.23	0.39	0.26
TAGLN2	**0.50**	0.38	0.04	0.10	0.23	0.22	0.16	0.42	0.23	0.21	0.37	0.26
RPS17	**0.51**	0.34	0.05	0.10	0.19	0.25	0.13	0.46	0.21	0.23	0.37	0.26
RPL8	**0.53**	0.38	0.04	0.02	0.14	0.22	0.17	0.49	0.19	0.21	0.41	0.26
PKP1	**0.52**	0.33	-0.09	0.13	0.27	0.19	0.19	0.49	0.19	0.17	0.36	0.25
RPS4X	**0.49**	0.36	0.06	0.01	0.22	0.22	0.17	0.46	0.19	0.22	0.37	0.25
RPL36	**0.53**	0.36	0.04	0.07	0.12	0.22	0.16	0.48	0.19	0.22	0.36	0.25
RPL5	**0.50**	0.34	0.06	0.08	0.17	0.23	0.14	0.43	0.22	0.21	0.36	0.25
PTMA	**0.48**	0.36	0.03	0.11	0.14	0.24	0.11	0.40	0.29	0.23	0.33	0.25
RPL24	**0.49**	0.37	0.03	0.06	0.18	0.21	0.22	0.44	0.19	0.18	0.34	0.25
RPL18	**0.49**	0.33	0.01	0.01	0.19	0.24	0.23	0.43	0.18	0.25	0.34	0.25
HLA.A	**0.48**	0.39	0.07	0.02	0.17	0.22	0.11	0.45	0.19	0.22	0.36	0.24
SFN	**0.57**	0.43	-0.12	0.11	0.16	0.17	0.25	0.53	0.07	0.11	0.40	0.24
GSTP1	**0.54**	0.38	-0.03	0.08	0.19	0.18	0.18	0.49	0.15	0.16	0.35	0.24
ACTB	**0.52**	0.41	0.08	0.11	0.06	0.18	0.14	0.47	0.14	0.17	0.37	0.24
ANXA2	**0.49**	0.39	0	0.12	0.15	0.18	0.19	0.44	0.17	0.18	0.34	0.24
TMSB10	**0.50**	0.40	0.08	0.07	0.10	0.21	0.12	0.44	0.18	0.20	0.37	0.24
ACTG1	**0.52**	0.41	0	0.08	0.14	0.20	0.20	0.46	0.12	0.15	0.38	0.24
PPIA	**0.49**	0.34	0	0.12	0.23	0.22	0.12	0.40	0.24	0.19	0.29	0.24

Supplementary Method 1

iStar

Due to variations in spatially resolved transcriptomics (SRT) technologies, H&E images from different datasets required specific rescaling. For the HER2+ ST dataset, the original pixel size was 2 μm with a spot radius of 50 μm; therefore, images were upscaled so that a 16×16 pixel patch represented a single cell. After rescaling, each spot contained approximately 125 cells. In the cSCC ST dataset, with a pixel size of 0.8 μm and a spot radius of 55 μm, images were upscaled, resulting in each spot containing about 150 cells. Conversely, the Visium-Kidney dataset had a pixel size of 0.22 μm and a spot radius of approximately 24 μm, requiring a downscaling, which led to spots containing around 30 cells.

Practical implementation of iStar requires consistency in image size, as well as image and spot resolutions, when training and testing across datasets. After rescaling to make a 16×16 patch represent a single cell, images from datasets with different resolutions vary significantly in size. Since image embeddings need to have the same dimension to be trained together, resizing can lead to information loss, requiring input images to have consistent dimensions. This requirement posed challenges when attempting to include TCGA and Visium-HER2+ as external validation data or perform cross-study analyses between the two breast cancer data Visium-Hercep-Test2+ and Visium-HER2+. For the HER2+ ST dataset, the upscaled image embeddings require a large amount of computational memory, making it challenging to train on multiple images simultaneously. We performed a subset evaluation for iStar by using only the first image slide from each training patient and predicting on the first slide from each test patient within each fold. An exception was made for patient G, as tissue annotation is available for the second slide.
